# Racism and the Social Defeat Hypothesis of Psychosis

**DOI:** 10.1192/j.eurpsy.2022.119

**Published:** 2022-09-01

**Authors:** J.-P. Selten, F. Termorshuizen

**Affiliations:** 1 Maastricht University, School For Mental Health And Neuroscience, Maastricht, Netherlands; 2 GGZ Rivierduinen, Fact-teams, Leiden, Netherlands

## Abstract

**Introduction:**

The social defeat hypothesis of psychosis posits that an outsider status or subordinate position is a common denominator of several psychosis risk factors, leads to elevated striatal dopamine function and thereby to an increased risk for the disorder. The purpose of this paper is to examine whether this hypothesis offers a good explanation for the increased psychosis risk for migrants from developing countries. Method: Review of literature. Results: The outsider status and subordinate position of many migrants from developing countries in Europe, and of Africans in particular, is evident. It is also clear that racism contributes to this situation. An interpretation of racism as psychotogenic is supported by findings of increased risks for other discriminated groups, such as homosexuals, individuals with a low IQ or a hearing impairment. The single study that examined dopamine function in migrants reported indeed elevated stress-induced dopamine release and dopamine synthesis capacity (Egerton et al., 2017). Recently, we examined the time course of the RR of treatment for psychosis for all migrants to the Netherlands (Termorshuizen & Selten, submitted). Contrary to our expectations, the RR for migrants from sub-Saharan Africa was already increased in the year of arrival (RR=2.5; 2.0-3.1), particularly for those aged 10-20 years (RR=6.1; 2.9-12.6). Conclusion: The social defeat hypothesis accords well with a psychotogenic effect of racism, but replications of the dopamine study are needed. The finding of an increased risk for Africans in the year of arrival suggests that racism is not the single cause of their increased risk.

**Disclosure:**

No significant relationships.

